# Defining features of hereditary lobular breast cancer due to *CDH1* with magnetic resonance imaging and tumor characteristics

**DOI:** 10.1038/s41523-023-00585-4

**Published:** 2023-09-27

**Authors:** Lauren A. Gamble, Paul H. McClelland, Martha E. Teke, Sarah G. Samaranayake, Paul Juneau, Amber L. Famiglietti, Andrew M. Blakely, Bernadette Redd, Jeremy L. Davis

**Affiliations:** 1 Surgical Oncology Program, Center for Cancer Research, National Cancer Institute, National Institutes of Health, Bethesda, MD USA; 2 Division of Library Services, Office of Research Services, National Institutes of Health, Bethesda, MD USA; 3 Radiology and Imaging Sciences, National Institutes of Health Clinical Center, Bethesda, MD USA

**Keywords:** Breast cancer, Cancer imaging

## Abstract

Women with germline pathogenic variants in *CDH1*, which encodes E-cadherin protein, are at increased lifetime risk of invasive lobular carcinoma (ILC). The associated tumor characteristics of hereditary lobular breast carcinoma (HLBC) in this high-risk population are not well-known. A single-center prospective cohort study was conducted to determine the imaging and pathologic features of HLBC compared to population-based ILC using Surveillance, Epidemiology, and End Results (SEER) data. One hundred fifty-eight women with *CDH1* variants were evaluated, of whom 48 (30%) also had an ILC diagnosis. The median age at *CDH1* diagnosis was 45 years [interquartile range, IQR 34–57 years] whereas the median age at diagnosis of *CDH1* with concomitant ILC (HLBC) was 53 [IQR 45–62] years. Among women with HLBC, 83% (40/48) were identified with *CDH1* mutation after diagnosis of ILC. Among 76 women (48%, 76/158) undergoing surveillance for ILC with breast magnetic resonance imaging (MRI), 29% (22/76) had an abnormal MRI result with available biopsy data for comparison. MRI detected ILC in 7 out of 8 biopsy-confirmed cases, corresponding with high sensitivity (88%), specificity (75%), and negative predictive value (98%); however, false-positive and false-discovery rates were elevated also (25% and 68%, respectively). HLBC was most frequently diagnosed at age 40–49 years (44%, 21/48), significantly younger than the common age of diagnosis of ILC in SEER general population data (most frequent age range 60–69 years, 28%; *p* < 0.001). HLBC tumors were smaller than SEER-documented ILC tumors (median 1.40 vs. 2.00 cm; *p* = 0.002) and had a higher incidence of background lobular carcinoma in situ (88% vs. 1%; *p* < 0.001) as well as progesterone receptor positivity (95% vs. 81%, *p* = 0.032). These findings suggest that HLBC is often detected via conventional screening methods as an early-stage hormone receptor-positive tumor, thus the clinical benefit of intensive screening with MRI may be limited to a subset of women with germline *CDH1* variants.

## Introduction

Invasive lobular carcinoma (ILC) accounts for 10–15% of all breast cancer cases and approximately 6% of incident ILC is associated with pathogenic germline variants^1–3^. In histopathologic analysis, ILC is most often characterized by infiltrating, poorly cohesive tumor cells with poor expression of E-cadherin, a cell adhesion protein encoded by the *CDH1* gene^2,4,5^. As such, pathogenic germline variants of *CDH1* have been associated with up to a 55% lifetime risk of ILC, which is most commonly attributed to this downstream loss of E-cadherin function^6^. In practice, loss-of-function *CDH1* variants are more commonly associated with hereditary diffuse gastric cancer (HDGC) syndrome^6,7^, which occurs both with and without concomitant ILC; to delineate between the two syndromes, families with a history of ILC and no history of diffuse gastric cancer are typically referred to as having hereditary lobular breast cancer (HLBC) specifically^5,8,9^. Despite this categorization, a thorough evaluation of families with germline *CDH1* pathogenic or likely pathogenic (P/LP) variants has indicated that HLBC is unlikely to comprise a distinct cancer syndrome but rather is part of the greater spectrum of diseases associated with *CDH1* mutations^6,10^. Therefore, in the absence of diffuse gastric cancer, germline genetic testing for *CDH1* mutations is recommended for individuals in the setting of early-onset ILC (age < 45 years), bilateral ILC (age <70 years), or extensive family history of ILC^1,2,8^.

Lobular carcinomas invade breast tissue linearly or in a single file, consistent with an infiltrative growth pattern. Accordingly, detection of ILC is challenging given that most tumors appear isodense, irregularly shaped, and devoid of microcalcifications on standard screening mammography (sensitivity 57–81%)^2,5,8,11,12^. Breast magnetic resonance imaging (MRI), when performed in conjunction with mammography, offers a more in-depth analysis of breast tissue and is the supplemental imaging modality of choice for women at high risk for breast cancer, with an overall screening sensitivity of 83–100% across various breast cancer types^5,13^. Screening with breast MRI is therefore recommended in high-risk patients with *CDH1* P/LP variants, with current consensus guidelines from the International Gastric Cancer Linkage Consortium (IGCLC) suggesting that patients should be routinely screened with annual breast MRI between 30–50 years of age, and potentially for longer^8^. These guidelines provide a framework for HLBC screening among *CDH1* P/LP mutation carriers, but evidence in support of these recommendations borrows from similar guidelines for *BRCA* mutation carriers, who are more prevalent than *CDH1* mutation carriers (0.2% vs. 0.06% of the general population) and more frequently develop invasive ductal carcinoma (IDC) rather than ILC^8,14,15^. As a result, while routine breast MRI is commonly employed to detect ILC in patients with *CDH1* mutations, its effectiveness as a screening modality has yet to be empirically verified in this unique population.

To this end, this study seeks to characterize breast MRI and pathologic findings specific to HLBC in a large cohort of women with germline *CDH1* P/LP variants. Particular attention is drawn to the unique features of this cancer with a complementary comparison to ILC in the general population to inform clinical guidance for cancer surveillance and risk reduction.

## Results

### Demographics and attributes of women with *CDH1* pathogenic variants

**Fig. 1:**
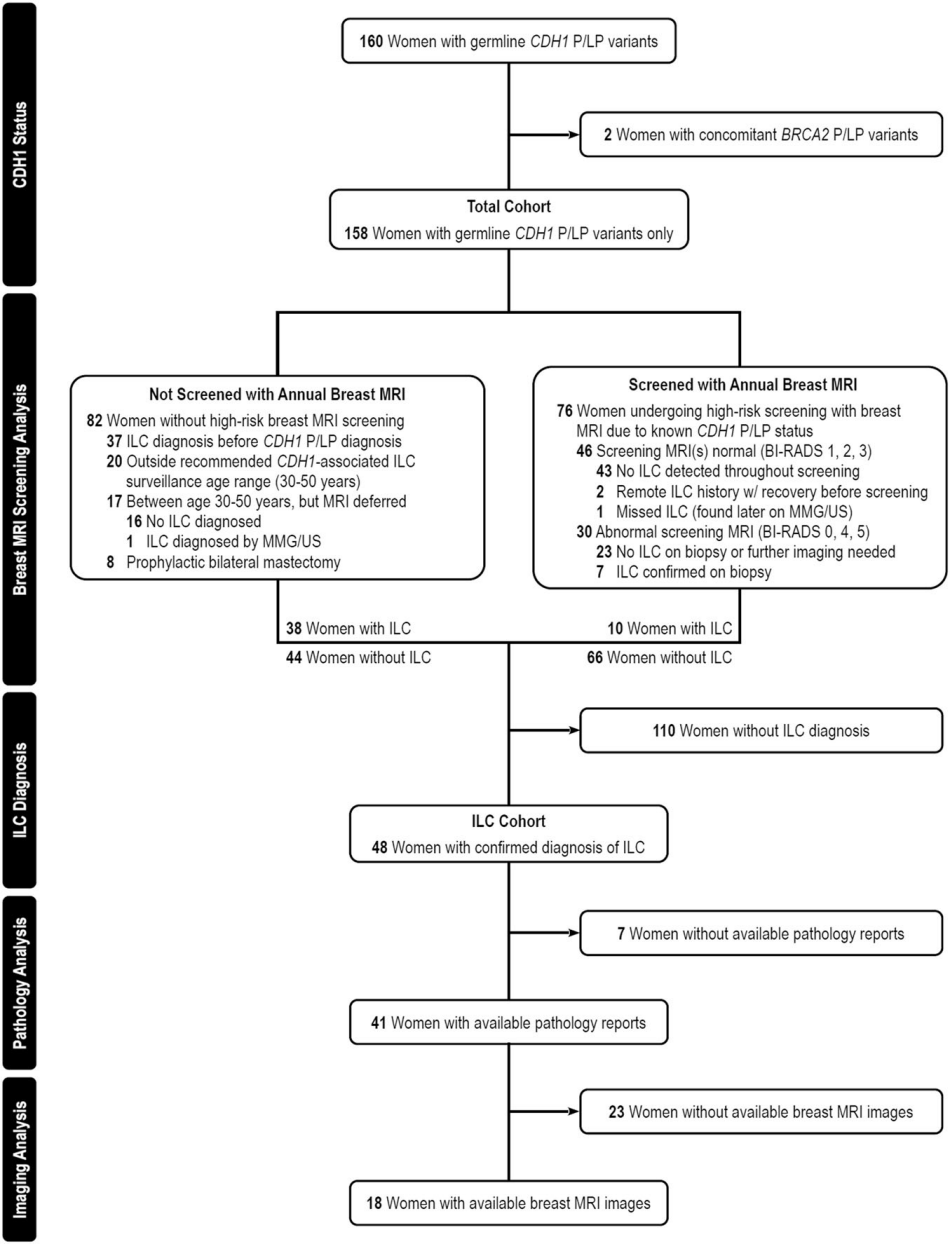
Flow diagram of women with *CDH1* variants, with and without ILC diagnosis. Approximately half of the individuals (48%, 76/158) in the study underwent recommended annual screening with breast MRI. Many women were diagnosed with ILC prior to diagnosis of a germline *CDH1* variant (83%, 40/48).

Over the five-year study period, 160 women with germline P/LP *CDH1* variants were documented. Two women were excluded for concomitant *BRCA2* pathogenic variant status, with 158 remaining eligible for analysis. From this main cohort of 158 women, two subcohorts were defined based on the presence of outpatient breast MRI screening data (“breast MRI screening” cohort, 76 women) and/or diagnosis of ILC (“ILC” cohort, 48 patients, Fig. 1).

In the main, 158-patient cohort, women were predominantly white (93%, 147/158) with 77% (121/158) reporting a family history of breast cancer and 78% (123/158) reporting a family history of gastric cancer (Table 1). These patients comprised 102 distinct lineages with 68 known families. The median age at *CDH1* diagnosis was 45 [interquartile range, IQR 34–57] years. Approximately one-third of *CDH1* variants were nonsense type (34%, 53/158), and mutations were most frequently located in the cadherin 4 domain (22%, 34/158).

**Table 1:** Demographics, family cancer history, and *CDH1* genotype characteristics.

	Total	Breast MRI screening			Malignancy
Characteristic	All women with *CDH1* (*n* = 158)	Not screened with breast MRI (*n* = 82)	Screened with breast MRI (*n* = 76)	*p*-value	Women with ILC (*n* = 48)
Age at *CDH1* diagnosis, median [Q1–Q3], years	45 [34–57]	47 [33–58]	44 [36–54]	0.686	53.0 [45–62]
Lineages, *n*	102	53	61		39
Race				0.543	
White, *n* (%)	147 (93.0)	76 (92.7)	71 (93.4)		46 (95.8)
Black, *n* (%)	4 (2.5)	2 (2.4)	2 (2.6)		1 (2.1)
Asian, *n* (%)	2 (1.3)	2 (2.4)	0 (0.0)		1 (2.1)
Other, *n* (%)	5 (3.2)	2 (2.4)	3 (3.9)		0 (0.0)
Family History					
Breast cancer, *n* (%)	121 (76.6)	62 (75.6)	59 (77.6)	0.764	37 (77.1)
Gastric cancer, *n* (%)	123 (77.8)	61 (74.4)	62 (81.6)	0.277	35 (72.9)
Genotype					
Variant type				0.444	
Deletion, *n* (%)	12 (7.6)	5 (6.1)	7 (9.2)		4 (8.3)
Frameshift, *n* (%)	29 (18.4)	13 (15.9)	16 (21.1)		9 (18.8)
Missense, *n* (%)	22 (13.9)	15 (18.3)	7 (9.2)		8 (16.7)
Nonsense, *n* (%)	53 (33.5)	26 (31.7)	27 (35.5)		17 (35.4)
Splice Site (canonical), *n* (%)	42 (26.6)	23 (28.0)	19 (25.0)		10 (20.8)
Variant location				0.427	
All, *n* (%)	3 (1.9)	2 (2.4)	1 (1.3)		2 (4.2)
PRE, *n* (%)	9 (5.7)	6 (7.3)	3 (3.9)		1 (2.1)
PRO, *n* (%)	15 (9.5)	5 (6.1)	10 (13.2)		6 (12.5)
Cadherin 1, *n* (%)	21 (13.3)^a^	11 (13.4)^b^	10 (13.2)^b^		5 (10.4)
Cadherin 2, *n* (%)	15 (9.5)	8 (9.8)	7 (9.2)		4 (8.3)
Cadherin 3, *n* (%)	7 (4.4)	5 (6.1)	2 (2.6)		4 (8.3)
Cadherin 4, *n* (%)	34 (21.5)^c^	20 (24.4)^d^	14 (18.4)^d^		8 (16.7)^d^
Cadherin 5, *n* (%)	28 (17.7)	14 (17.1)	14 (18.4)		10 (20.8)
Transmembrane, *n* (%)	7 (4.4)	5 (6.1)	2 (2.6)		4 (8.3)
Cytoplasmic, *n* (%)	19 (12.0)	6 (7.3)	13 (17.1)		4 (8.3)

Continuous data described as median with IQR [IQR, Q1–Q3]; these averages were compared via Mann–Whitney *U-*test. Categorical variables were assessed via frequencies and proportions and compared via *χ*^2^ test.

*HLBC* hereditary lobular breast cancer, *IQR* interquartile range.

^a^2 variants located in the PRO-EC1 linker region.

^b^1 variant located in the PRO-EC1 linker region.

^c^2 variants located in the EC3-EC4 linker region.

^d^1 variant located in the EC3-EC4 linker region.

### Screening breast magnetic resonance findings in women with *CDH1* P/LP variants

Of the total cohort of women with *CDH1* P/LP variants, nearly half (48%, 76/158) had high-risk screening breast MRI data available for review (Fig. 1). Post hoc breast MRIs, such as those used for surveillance after treatment (if applicable), were not considered for inclusion in this group. Notably, two women included in this screening cohort had a prior remote history of ILC (4 and 20 years prior to *CDH1* mutation diagnosis, respectively); however, these individuals were still considered to participate in high-risk breast MRI screening since both had completed treatment, neither underwent mastectomies and screening breast MRI dates for both corresponded with *CDH1* mutation diagnosis, not previous malignancy.

Among the 76 women screened with breast MRI, the median age of initial screening was 44 [36–54] years, with most women having their first breast MRI performed in the fourth or fifth decade of life (Table 1). These age ranges aligned with the median age of *CDH1* mutation diagnosis in this cohort (44 [36–54] years), with more than half undergoing their first breast MRI within one year after genetic testing (41/76, 54%). When compared with the current breast MRI screening recommendations for high-risk *CDH1* P/LP variant carriers^8^, 8/76 (11%) women were adherent and had their first breast MRI by age 30, whereas 44/76 (59%) had their first breast MRI between ages 30 and 50, and 24/76 (32%) had their first breast MRI after age 50 (Supplementary Fig. 1). While most patients underwent multiple rounds of breast MRI as part of their screening regimen (43/76, 57%), a substantial minority had only undergone a single breast MRI at the time of analysis (33/76, 43%). For the remaining 82/158 (52%) patients not screened with breast MRI, 37 (45%) were diagnosed with ILC prior to genetic testing for *CDH1*, 20 (24%) were aged outside the recommended breast MRI screening window (<30 or >50 years), 17 (21%) had no prior surveillance imaging or reports were unobtainable, and 8 (10%) reported bilateral risk-reducing mastectomy without a preoperative breast MRI. There were no statistically significant differences in demographics, family cancer histories, and *CDH1* mutation genotype between screened and non-screened cohorts.

Women undergoing routine breast MRI were most frequently reported as having benign findings on imaging throughout the screening period (Breast Imaging Reporting and Data System (BI-RADS) 1, 2, 3; 61%, 46/76), whereas 39% (30/76) of women in this cohort had an instance of abnormal breast MRI or incomplete imaging (BI-RADS 0, 4, 5). In instances of incomplete imaging (BI-RADS 0), a second-look ultrasound was routinely recommended to clarify results. Among the 30 women with abnormal or incomplete imaging, subsequent diagnostic workup was available for 22 individuals. Eight of these women were found to have pathologically confirmed ILC: five women with BI-RADS 4, two with BI-RADS 0, and one with a false-negative BI-RADS 1 on breast MRI. For the seven women diagnosed with *CDH1*-associated ILC via screening breast MRI, the median age at diagnosis was 46 [44.5–55.5], and all had negative screening with breast mammogram and/or ultrasound prior to screening with breast MRI. For the one patient with a false-negative screening breast MRI, a 0.6-cm mass (BI-RADS 4) was found on follow-up mammography 6 months after breast MRI screening at age 35. This was confirmed as ILC on biopsy and reimaged with diagnostic breast MRI thereafter (BI-RADS 6). When compared with available follow-up biopsy data, these screening statistics corresponded with a sensitivity of 88% (7/8), a specificity of 75% (45/60), a positive predictive value (precision) of 32% (7/22), a negative predictive value of 97.8% (45/46), and an overall MRI detection rate of 4% (7/166). The false-positive and false-discovery rates in this cohort were 25% and 68%, respectively (Supplementary Table 1).

### Hereditary lobular breast cancer on magnetic resonance imaging

**Fig. 2:**
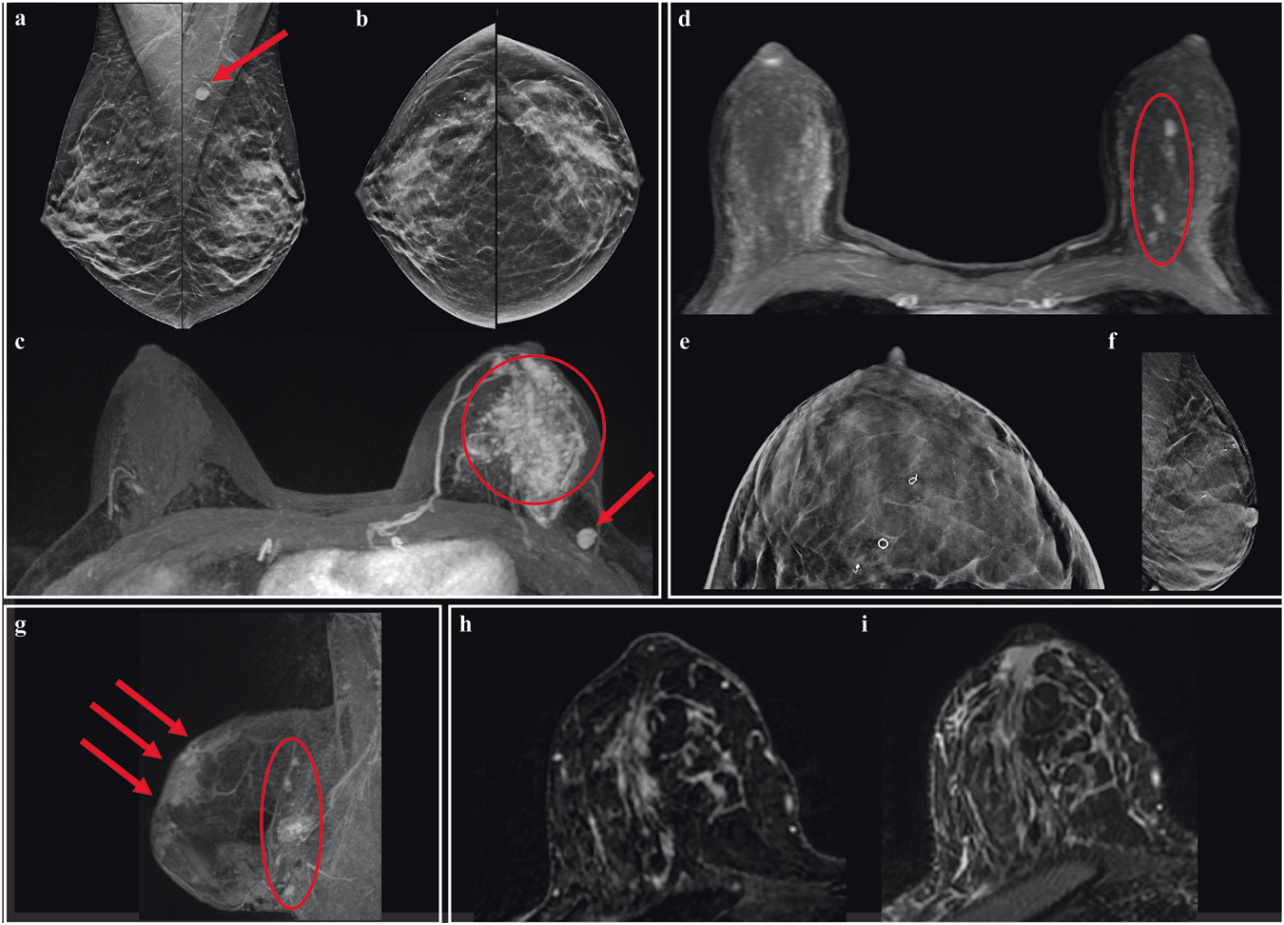
Breast imaging in women with hereditary lobular breast cancer. Patients with HLBC undergoing diagnostic breast MRI either due to a palpable abnormality or an abnormality detected on a screening mammogram generally had a significantly larger tumor burden at the time of diagnosis compared with patients whose breast cancer was diagnosed on screening breast MRI. In a 47-year-old female with an unknown germline *CDH1* variant presenting with a palpable abnormality, a mammogram demonstrated subtle asymmetry in the left breast on both mediolateral (**a**) and craniocaudal (**b**) views. Follow-up imaging with breast MRI axial MIP (maximum intensity projection) (**c**) showed an approximate 7-cm region of non-mass enhancement (red circle) and a prominent left axillary node with rounded morphology, which was also seen on the original mammogram (red arrow). Final pathology demonstrated a 7-cm mixed ductal/lobular carcinoma in both the left upper outer quadrant and the left lower outer quadrant. In contrast, a 45-year-old woman with known germline *CDH1* P/LP variant received a recommended screening breast MRI (**d**), which demonstrated multiple small enhancing masses in the left breast, seen on an axial MIP image (red circle). Follow-up ultrasound and biopsy with clip placement confirmed two sites of malignancy in the left breast. Post-biopsy mammogram assessing clip placement sites demonstrated dense breast tissue without an obvious mass at biopsy sites on either the mediolateral (**e**) or craniocaudal (**f**) views. The final pathology for this individual demonstrated a 1.1-cm invasive lobular carcinoma. To showcase MRI detection of uncommon features, (**g**) depicts a 66-year-old female with known germline *CDH1* P/LP variant originally diagnosed with a 6-cm invasive lobular breast carcinoma at age 65 who was treated with bilateral mastectomy and systemic chemotherapy but developed skin changes the following year; follow-up breast MRI demonstrated several prominent intramammary and axillary lymph nodes (red circle, sagittal view) with subdermal enhancing abnormalities and skin thickening signaling recurrence of disease (red arrows). Last, techniques such as STIR may further assist in the detection of tumors on breast MRI, as seen in Figures **h**, **i**: in this case, a 52-year-old female with a known germline *CDH1* variant demonstrated an ill-defined large infiltrating tumor in the right breast on post-contrast axial MRI (**h**), correlating with parenchymal edema on corresponding STIR sequence (**i**).

Among women with both a biopsy-confirmed diagnosis of ILC and corresponding breast MRI, 18 (38%) had complete breast screening or diagnostic MRI images available, which allowed for the characterization of *CDH1*-associated ILC (Table 2). For all 18 available studies, background parenchymal enhancement was either mild or minimal, and fibroglandular density was variable between patients but did not limit qualitative analysis. In general, tumors appeared larger on breast MRI than on mammogram or ultrasound, and breast MRI revealed more extensive disease than initially appreciated via other modalities (Fig. 2a–f). Invasive lobular carcinoma on breast MRI demonstrated a combination of discrete mass-like enhancement (78%, 14/18) and non-mass enhancement (88%, 15/17). Most masses further featured non-circumscribed borders (85%, 11/13) and/or internal enhancement (93%, 13/14). Non-mass enhancement was frequently regional in distribution (53%, 8/15) with a clumped morphology (80%, 12/15). A minority of patients (28%, 5/18) had other findings, including skin or nipple retraction (3/5, 60%), axillary adenopathy (3/5, 60%), and architectural distortion (2/5, 40%, Fig. 2g). Fat-suppressed T2 and short tau inversion recovery (STIR) sequences were useful in identifying breast tissue edema and skin thickening (Fig. 2h, i).

**Table 2:** Breast MRI features of pathologically confirmed HLBC.

Feature	Women with ILC and breast MRI data^a^ (*n* = 18)
Size^b^, median [Q1–Q3], cm	5.0 [2.1–7.4]
Discrete mass, *n* (%)	14/18 (77.8)
*Shape*	14/14 (100.0)
Round, *n* (%)	2/14 (14.3)
Irregular, *n* (%)	11/14 (78.5)
Other^c^, *n* (%)	1/14 (7.1)
*Margin, n (%)*	13/14 (92.9)
Circumscribed, *n* (%)	2/13 (15.4)
Round, *n* (%)	1/2 (50.0)
Irregular, *n* (%)	1/2 (50.0)
Non-circumscribed, *n* (%)	11/13 (84.6)
Irregular, *n* (%)	6/11 (54.5)
Spiculated, *n* (%)	5/11 (45.5)
*Internal enhancement, n* (%)	13/14 (92.9)
Homogenous, *n* (%)	8/13 (61.5)
Heterogeneous, *n* (%)	5/13 (38.5)
Non-mass enhancement, *n* (%)	15/17 (88.2)
*Distribution, n (%)*	15/15 (100.0)
Regional, *n* (%)	8/15 (53.3)
Focal, *n* (%)	5/15 (33.3)
Linear, *n* (%)	1/15 (6.7)
Multiple regions, *n* (%)	1/15 (6.7)
*Internal enhancement, n (%)*	15/15 (100.0)
Homogenous, *n* (%)	0/15 (0.0)
Heterogeneous, *n* (%)	3/15 (20.0)
Clumped, *n* (%)	12/15 (80.0)
Associated features, *n* (%)	5/18 (27.8)
Skin or nipple retraction, *n* (%)	3/5 (60.0)
Axillary adenopathy, *n* (%)	3/5 (60.0)
Architectural distortion, *n* (%)	2/5 (40.0)

*HLBC* hereditary lobular breast cancer, *IQR* interquartile range.

^a^Women with ILC who had breast MRI available for radiographic assessment

^b^Size of largest single dimension based on MRI assessment

^c^Too small to characterize (obscured by clip).

### Diagnosis and histopathology of hereditary lobular breast cancer due to *CDH1*

Forty-eight women (30%, 48/158) were diagnosed with or had a history of ILC, of whom 40 (83%) were identified with a *CDH1* P/LP variant because of their ILC diagnosis: 38 of these individuals were diagnosed using methods outside of high-risk annual breast MRI screening, and two individuals originated from the breast MRI screening cohort but had a prior remote diagnosis of ILC using non-MRI imaging (Fig. 1). Among these 40 patients, a combination of mammogram and ultrasound was the most common method of diagnosing ILC (36/40, 90%), followed by other/unknown methods (3/40, 7.5%) or rarely computed tomography (1/40, 2.5%). For all 48 women with HLBC, the median age of diagnosis for *CDH1* P/LP germline mutation and ILC was 53 [45–62] and 47 [44–56] years respectively, and 46% (22/48) of women were diagnosed with ILC after age 50 years (Table 1 and Supplementary Table 2).

Diagnostic pathology reports were available for 85% (41/48) of women diagnosed with ILC due to *CDH1*. Most of these cases (85%, 35/41) revealed pure lobular carcinoma, and 6 (15%, 6/41) had mixed-type invasive carcinoma (Supplementary Table 2). Four women had bilateral carcinomas at diagnosis (10%), and three women had metachronous breast cancer (7%). Resected breast cancers had a median size of 1.4 [0.8–1.8] cm, with 76% (28/37) classified as pathologic stage T1 (≤2 cm in size). Tumors were most frequently intermediate nuclear grade (grade 2, 16/33, 48%) and intermediate histologic grade (grade 2, 19/30, 63%). Ninety-five percent (37/39) of tumors were estrogen receptor-positive (ER+), 95% (36/38) were progesterone receptor-positive (PR+), and 8% (3/36) were HER2/neu-positive (HER2+). Additional findings such as atypical lobular hyperplasia (ALH), intermediate- or high-level Ki-67 expression, and lymphovascular invasion were also noted in some tumors, although reporting of these features was inconsistent across pathology records (10/10, 12/16, and 4/23, respectively).

Clinically, most tumors did not have regional lymph node involvement at the time of diagnosis (26/36, 72%). Applying American Joint Committee on Cancer/Union for International Cancer Control (AJCC/UICC) 8^th^ edition pathologic prognostic staging guidelines to patients with complete or *de facto* complete criteria (TNM stage, histologic grade, ER/PR/HER2 status)^16^, the staging distribution among patients with HLBC was 28/34 (82%) IA, 2/34 (6%) IB, 1/34 (3%) IIA, 1/34 (3%) IIB, and 2/34 (6%) IIIA.

### Hereditary lobular breast cancer compared to ILC in the general population

**Fig. 3:**
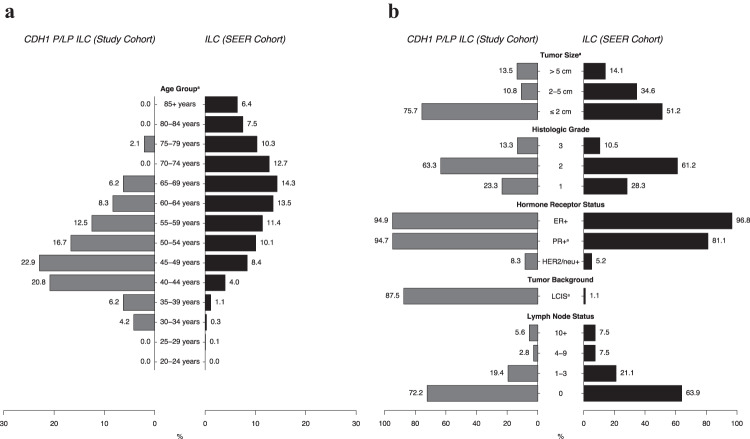
Comparison of hereditary lobular breast cancer and ILC in the general population. Bar graphs comparing (**a**) age of diagnosis and (**b**) pathologic findings for women with HLBC due to *CDH1* P/LP variants and women with ILC from SEER data. On average, women with HLBC were younger at the time of diagnosis, and primary tumors were smaller in size with a higher incidence of concomitant LCIS.

SEER data were used to compare women with germline *CDH1*-associated ILC to rates of ILC in the general population (Fig. 3 and Supplementary Table 2). Women with HLBC were diagnosed at a younger age (*p* < 0.001), with most cases (44%, 21/48) diagnosed between 40 and 49 years of age. In contrast, ILC cases in the general population were most frequently discovered during the sixth, seventh, and eighth decades of life with an even distribution (21%, 28%, and 23%, respectively). Median tumor size for HLBC in this study was smaller compared to population-based ILC (1.4 [0.8–1.8] vs. 2.0 [1.2–3.5] cm, *p* = 0.002), with HLBC having a higher proportion of stage T1 tumors (76% vs. 51%, *p* = 0.006). Histologic grading distribution was similar between the two groups (most commonly grade 2, 63% vs. 61%, *p* = 0.776), although HLBC was more often reported as mixed histotype (14% vs. 0.6%, *p* < 0.001) and in a background of lobular carcinoma in situ (LCIS, 88% vs. 1%, *p* < 0.001). With regard to hormone receptors, HLBC tumors were found to have a higher frequency of PR+ status compared to ILC tumors in the general population (95% vs 81%, *p* = 0.032), whereas rates of ER+ and HER2+ status were roughly equivalent. Moreover, there was no significant difference in positive lymph node status at diagnosis between HLBC and ILC in the general population (28% vs. 36%, *p* = 0.301).

## Discussion

This study demonstrates the complexities of breast cancer risk associated with HLBC due to germline *CDH1* P/LP variants, including both the natural history of this disease as well as the demographic, pathologic, and radiographic characteristics seen in this patient population. Notably, this study establishes that HLBC most frequently occurs during the fifth decade of life for women with *CDH1* P/LP variants, which is significantly younger than ILC diagnosis in the general population. In addition, this analysis shows that diagnosis of *CDH1*-associated ILC often precedes diagnosis of *CDH1* germline mutations, meaning that many individuals with *CDH1* P/LP germline mutations do not undergo recommended high-risk screening with annual breast MRI but rather have ILC diagnosed incidentally or via normal-risk screening with mammogram or ultrasound. On imaging, *CDH1*-associated ILC shares many characteristics with conventional ILC, including lepidic, infiltrative, and irregular growth with both mass and non-mass enhancement. When employed as a screening tool, breast MRI is effective at detecting these tumors with high sensitivity (88%), specificity (75%), and negative predictive value (98%); however, these detection rates are accompanied by elevated false-positive and false-discovery rates (25% and 68%, respectively). On pathological analysis, *CDH1*-associated ILC tumors are generally smaller than ILC in the general population, with higher rates of PR expression, mixed histotype, and background LCIS but similar grading distribution, frequency of ER/HER2 expression, and lymph node involvement. Taken together, these results underscore the need for early detection of *CDH1* germline mutations to allow for timely initiation of high-risk breast cancer screening, for which breast MRI appears to be effective although beset by false-positive results.

Despite the current recommendation of initiating annual breast MRI by age 30 for women with germline *CDH1* P/LP variants^8^, the majority of patients with HLBC in this analysis were diagnosed outside of this high-risk screening paradigm. Among 48 patients with *CDH1*-associated ILC, 40 (83%) were diagnosed with ILC prior to the discovery of their *CDH1* P/LP mutation status and thus had successful detection of their malignancy without the use of breast MRI screening. Moreover, among those proactively screened with annual breast MRI, only 11% (8/76) started screening by age 30 as recommended, with initial screening breast MRIs occurring at a median age of 44 years, often directly after diagnosis of *CDH1* P/LP mutations. These observations reflect an ongoing challenge in cancer risk assessment for women with germline *CDH1* P/LP variants: since individual *CDH1* genetic testing criteria are largely centered around *post hoc* manifestations of disease (i.e., diffuse gastric cancer and/or lobular breast cancer), preemptive screening is almost entirely reliant on family history, which can be difficult to ascertain due to the rarity of *CDH1* mutations, patient recall bias, and other factors^8,17,18^. To mitigate this problem, some studies have advocated for regular inclusion of *CDH1* on multigene panel testing, which may detect unexpected *CDH1* P/LP variants in a wider variety of patients with more diverse family histories; as these tests become cheaper and more widely available, the number of patients benefiting from early screening with breast MRI may increase accordingly^15,19^. Alternatively, population-based risk stratification tools such as the Gail model may also theoretically be used to estimate the likelihood of developing ILC in certain populations, although some studies have cautioned against using these models in the setting of syndromic breast cancers^20,21^. Considering that other factors such as variant-specific disease and *CDH1* mutation penetrance likely also play a role in disease manifestation, early and precise determination of *CDH1* mutation status remains an essential component of subsequent cancer detection in these patients, and screening protocols may become increasingly individualized as additional information regarding *CDH1* P/LP mutations comes to light^6–8,17^.

When used as a screening tool for *CDH1*-associated ILC, breast MRI proved to be a sensitive method of detecting malignancy in this analysis, with a sensitivity of 88%, a specificity of 75%, and an overall detection rate of 4%. This detection rate is comparable to that observed in women with other hereditary breast cancer syndromes such as *BRCA, PTEN*, and *TP53*, who are also often routinely screened with breast MRI (aggregate 2.98%; 30 per 1000 MRI tests)^22^. In actuality, much of the data surrounding the utility of screening breast MRI originates from *BRCA* pathologic variant carriers, for whom breast MRI has demonstrated superior sensitivity for multiple types of breast cancers compared to mammogram or ultrasound alone (typically >90%)^23–25^. Moreover, when considering that many high-risk patients must initiate screening at a young age, additional advantages of breast MRI such as high sensitivity in dense breasts become increasingly relevant^8,26–28^. As such, breast MRI has been widely adopted as an adjunct to mammography in long-term high-risk breast cancer screening^29–34^, with some evidence even suggesting no additional screening benefit with the use of mammography in addition to MRI in women under the age of 40 with *BRCA* pathogenic variants^35^. However, despite these observed benefits in the *BRCA* pathogenic variant population, the utility of routine breast MRI among *CDH1* P/LP variant carriers is less clear. In the current analysis, screening breast MRI demonstrated a true detection benefit in a minority of patients with otherwise negative conventional mammogram/ultrasound screening (7/76, 9.2%), whereas false-positive and false-discovery rates were high among the remainder of the cohort (25% and 68%, respectively). Falsely positive findings in the absence of meaningful clinical benefit may lead to increased screening burden via additional healthcare costs, unnecessary biopsies, and undesirable downstream effects^23,36,37^.

Because of its increased sensitivity, breast MRI has an important role as a diagnostic tool, possibly more so than a screening modality, among *CDH1* P/LP carriers who not only represent a young patient cohort but also classically present with infiltrative tumors such as ILC. Compared to mammography and ultrasound, breast MRI is shown to have an improved ability to estimate true tumor size while identifying complex features such as multifocality and multicentricity. Some studies have reported the discovery of additional occult disease in 10–60% of ILC cases with breast MRI after initial imaging with mammogram and ultrasound^28,38–41^. Additionally, standard mammography has been shown to be less sensitive for ILC than IDC, which may indicate potential uses for breast MRI in an adjunctive context when planning treatment of disease^42,43^. Therefore, breast MRI may still prove useful as part of a hybrid screening strategy for ILC in the *CDH1* P/LP variant carrier population, although more research is needed to quantify the benefits of such a strategy in this cohort.

In-depth analysis of breast MRI scans in patients with HLBC revealed several potentially useful imaging characteristics for the recognition of ILC. Although a dominant imaging pattern was not identified in this study group, cases of *CDH1*-associated ILC often demonstrated mass and non-mass enhancement with non-circumscribed, spiculated, and irregular borders, which corroborates the hormone receptor-positive and infiltrative nature of this disease^5,44,45^. Moreover, tumors often appeared much larger on breast MRI compared to concurrent mammography, ultrasound, or physical examination, which is common for breast MRI in most breast cancers but has been shown for ILC, in particular, to correlate closely with true tumor size on final surgical pathology^39,40^. For additional abnormalities such as breast edema and skin thickening, complementary fluid-sensitive MRI sequences such as fat-suppressed T2 and STIR were useful adjuncts in this analysis, and these techniques have been widely employed for ILC tumors in general to enhance results^39^. While not definitively diagnostic, broad genotype-phenotype correlations on imaging such as those seen in this analysis have occasionally been described in other high-risk populations: for example, *BRCA2* pathogenic variants have been associated with heterogeneous, irregular, and spiculated tumors, whereas *BRCA1* pathogenic variants have been associated with more rounded and circumscribed breast masses^46,47^. As for *CDH1*, there are over 150 known P/LP mutations of varying penetrance that span the entire gene, which may ultimately lead to heterogeneous findings on MRI regardless of umbrella P/LP variant status^2,48–50^. Thus, while larger imaging studies of *CDH1*-associated ILC may elucidate important diagnostic and potential phenotypic tumor characteristics, biopsy with histopathologic analysis will likely remain the diagnostic gold standard for these lesions.

Microscopically, both general ILC and *CDH1*-associated ILC classically appear as infiltrative, poorly cohesive tumors with cells arranged linearly or in single file, a growth pattern typically attributed to loss of calcium-dependent cell adhesion due to dysfunctional or absent E-cadherin expression^2,4,5,49^. In line with this, pathologic findings from risk-reducing mastectomies in *CDH1* variant carriers frequently report the presence of occult bilateral high-risk lesions and neoplastic foci including ALH, LCIS, and ILC^4,5,8,18,51^, all of which can be characteristically E-cadherin-negative^5,52^. To date, the exact relationship between E-cadherin dysfunction, premalignant ALH/LCIS, and malignant ILC is unclear: ALH and LCIS share many of the same genetic aberrations as ILC and are both considered nonobligate precursors of lobular breast cancer, but a definitive pathway to malignancy between these different lesions has yet to be established^9,53^. In fact, despite the common designation of LCIS as a premalignant lesion, coexisting LCIS and ILC have counterintuitively been associated with less aggressive disease compared to isolated ILC, demonstrating decreased nodal involvement, decreased locoregional spread, and improved disease-free survival, with particularly favorable outcomes seen in patients under 50 years of age^54,55^. Since most *CDH1*-associated ILC tumors in this analysis were diagnosed at younger ages with concomitant LCIS, these results may portend better breast outcomes for this current study cohort compared to the population at large. Moreover, the observation that *CDH1*-associated ILC tumors in this analysis were frequently hormone receptor-positive (ER + 95%, PR + 95%) introduces the possibility of endocrine therapy as a form of chemoprophylaxis in patients with *CDH1* P/LP mutations^4^.

Given the overall rarity of *CDH1* germline P/LP variant carriers and the scarcity of data for this cohort, this study has several inherent limitations. For one, much of the imaging and histopathologic analysis performed in this study was based on reports and images from outside institutions, which led to some heterogeneity in reported results as well as missing data when studies were unavailable. Several measures were taken to mitigate this issue, including limiting histopathologic analyses to key reportable features and using the BI-RADS scoring system to formalize breast MRI results. Moreover, when possible, raw images were preferentially obtained for review by a single expert radiologist with extensive experience in breast MRI (BR), with all findings standardized using the common BI-RADS lexicon. Another limitation in this study was the lack of proper comparators for breast MRI data: since concurrent mammogram and ultrasound images were not always available for breast MRI scans, direct comparison between breast MRI and other screening modalities was not possible for HLBC, and comparisons between HLBC and standard ILC on imaging were similarly not performed due to a lack of normalized ILC imaging data from the general population. Discounting comparative analysis, qualitative breast MRI analysis was also potentially influenced by the frequent presence of LCIS with ILC tumors, which may have led to the overestimation of tumor sizes due to the close resemblance in imaging. For portions of the study using SEER data, findings were limited by the extreme difference in cohort sizes (48 vs. 100,266), lack of coding of certain variables (e.g., nuclear grade, Ki-67 expression), and other inherent limitations of the SEER database^56,57^. Last, longitudinal follow-up after ILC diagnosis was not routinely performed in this analysis, which would allow for extended assessment of observed MRI detection rates compared to expected rates based on cancer penetrance estimates.

Hereditary lobular breast cancer most commonly presents as small, infiltrative, intermediate-grade, hormone-receptor-positive tumors that arise as early as the fourth decade of life and peak during the fifth and sixth decades of life in women with *CDH1* P/LP variants, significantly younger than invasive lobular carcinomas seen in the general population. These data demonstrate that for a majority of women with *CDH1* variants who develop ILC, these breast cancers are likely to come to clinical attention via conventional screening methods (e.g., without breast MRI), and are likely to be early stage with favorable receptor subtype. Accordingly, the benefit of intense screening with annual breast MRI may be somewhat diminished, as earlier diagnosis is unlikely to alter treatment or outcomes for those diagnosed with stage I disease regardless of screening modality. However, more than a quarter of this cohort presented with node-positive cancers, and several cases of ILC were mammographically occult and identified on screening MRI only, suggesting a potential benefit of earlier detection and enhanced screening in a subset of *CDH1* variant carriers. The optimal age at which to start and stop screening is still unknown. Notably, however, nearly half of the ILC cases in this cohort were diagnosed in women older than age 50, suggesting that MRI screening should be considered beyond this age. Ideally, the impact of high-risk screening with breast MRI on treatment and survival outcomes should be evaluated. Although these data likely will be difficult to obtain, longitudinal evaluation of an expanded cohort may help delineate a subset of women for whom enhanced screening may be warranted.

## Methods

### Study population

Female patients enrolled in a single-center, prospective natural history study of HDGC syndrome (NCT03030404) between October 2017 and January 2022 were considered for this analysis.

Individuals included in this analysis were carriers of a confirmed germline P/LP variant in the *CDH1* gene. Women were excluded if they had a family history of ILC and no germline genetic defect, a *CDH1* variant of uncertain significance, or other high-risk gene variants (e.g., *BRCA*, *PALB2*). Genetic testing was performed by laboratories adherent to the American College of Medical Genetics and the Association for Molecular Pathology guidelines for the interpretation of sequence variants. Demographic data, breast imaging and reports, breast pathology, detailed family pedigrees, and *CDH1* genotype were obtained for all eligible patients. All procedures performed in studies involving human participants were in accordance with the ethical standards of the Declaration of Helsinki, and all patients provided written informed consent to enroll in this study, which was approved by the Institutional Review Board of the National Institutes of Health.

Available breast imaging studies performed at outside centers were collected and reviewed by a board-certified radiologist (BR) with more than 25 years of experience in breast MRI. Breast MRI studies were categorized as either screening or diagnostic. Screening breast MRI exams were defined as those obtained in asymptomatic, high-risk patients, whereas diagnostic breast MRI exams were defined as those obtained for either a new diagnosis of breast cancer or acquired for additional evaluation of indeterminate findings on a preceding exam. The Philips Achieva 3 T MR system was utilized for imaging patients at the authors’ institution. Imaging findings were characterized by the Breast Imaging Reporting and Data System (BI-RADS), with scoring as follows: 0—further information or workup required; 1—negative; 2—benign findings; 3—probable benign finding (short follow-up interval required); 4—suspicious abnormality; 5—highly suggestive of malignancy; 6—known biopsy-proven malignancy^58^. In this study, patients with imaging features classified as BI-RADS 0, 4, or 5 were considered to have abnormal breast MRI findings.

Similarly, pathology reports from outside centers were collected and evaluated to verify the presence of invasive disease as well as other defining characteristics. In accordance with updated guidelines from the American Society of Clinical Oncology (ASCO) and the College of American Pathologists (CAP)^59^, positive estrogen or progesterone receptor status (ER+/PR+) was defined as samples with 1–100% of tumor nuclei staining positive for the receptor in question. Positive human epidermal growth factor receptor 2 status (HER2+) was defined as either frankly positive staining on immunohistochemistry (3+) or equivocal staining (2+) with corresponding positive reading on fluorescence in situ hybridization (FISH) analysis. Ki-67 proliferation index was categorized as low (<10%), intermediate (≥10%, <20%), or high (≥20%) to stratify patients into appropriate risk categories. Other pathologic findings were documented as described in the corresponding pathology reports.

To characterize ILC in the general population, data were queried from the Surveillance, Epidemiology, and End Results (SEER) Research Limited Field Data Incidence Database, which included 17 registries between 2000 and 2019 (November 2021 submission). The 2008 World Health Organization International Classification of Diseases for Oncology coding system, third revision (WHO ICD-O-3) was used to define pathology. Within the database, morphology code 8520 (“lobular carcinoma”) with corresponding breast primary location was used to define a diagnosis of primary ILC, and male sex was excluded. To homogenize results, age was divided into either 5- or 10-year intervals, and histologic grade was based on the Scarff-Bloom-Richardson (SBR) grading system adapted for SEER. The presence of concomitant LCIS was defined as the presence of an additional 8520 morphology code diagnosed at the same time as the primary code, with additional behavior designation corresponding with premalignancy (“in situ,” “borderline malignancy”). Other variables such as tumor size and receptor status were obtained directly from the database.

### Statistical analysis

Data were analyzed with Statistical Product and Service Solutions (SPSS®) (v25.0, IBM Corporation, Armonk, NY) and R statistical programming language (v4.2.2, R Foundation for Statistical Computing, Vienna, Austria, 31 October 2022). For continuous variables, data were described as medians with interquartile ranges [IQR, Q1–Q3]; these averages were compared via the Mann–Whitney *U*-test. Categorical variables were assessed via frequencies and proportions and were compared via *χ*^2^ test. All *p*-values were two-sided with a statistical significance evaluated at the 0.05 alpha level. A trajectory graph (Supplementary Fig. 1) was generated using a Statistical Analysis System (SAS^TM^) (v9.4, SAS Institute Inc., Cary, NC). Study approval was obtained from the National Institutes of Health Institutional Review Board.

### Reporting summary

Further information on research design is available in the Nature Research Reporting Summary linked to this article.

### Supplementary information

Supplementary Files

Reporting Summary

## Data Availability

Data generated in this study are available upon reasonable request from the corresponding author.

## Code availability

The code used for statistical calculations in this analysis is available upon reasonable request from the corresponding author.
