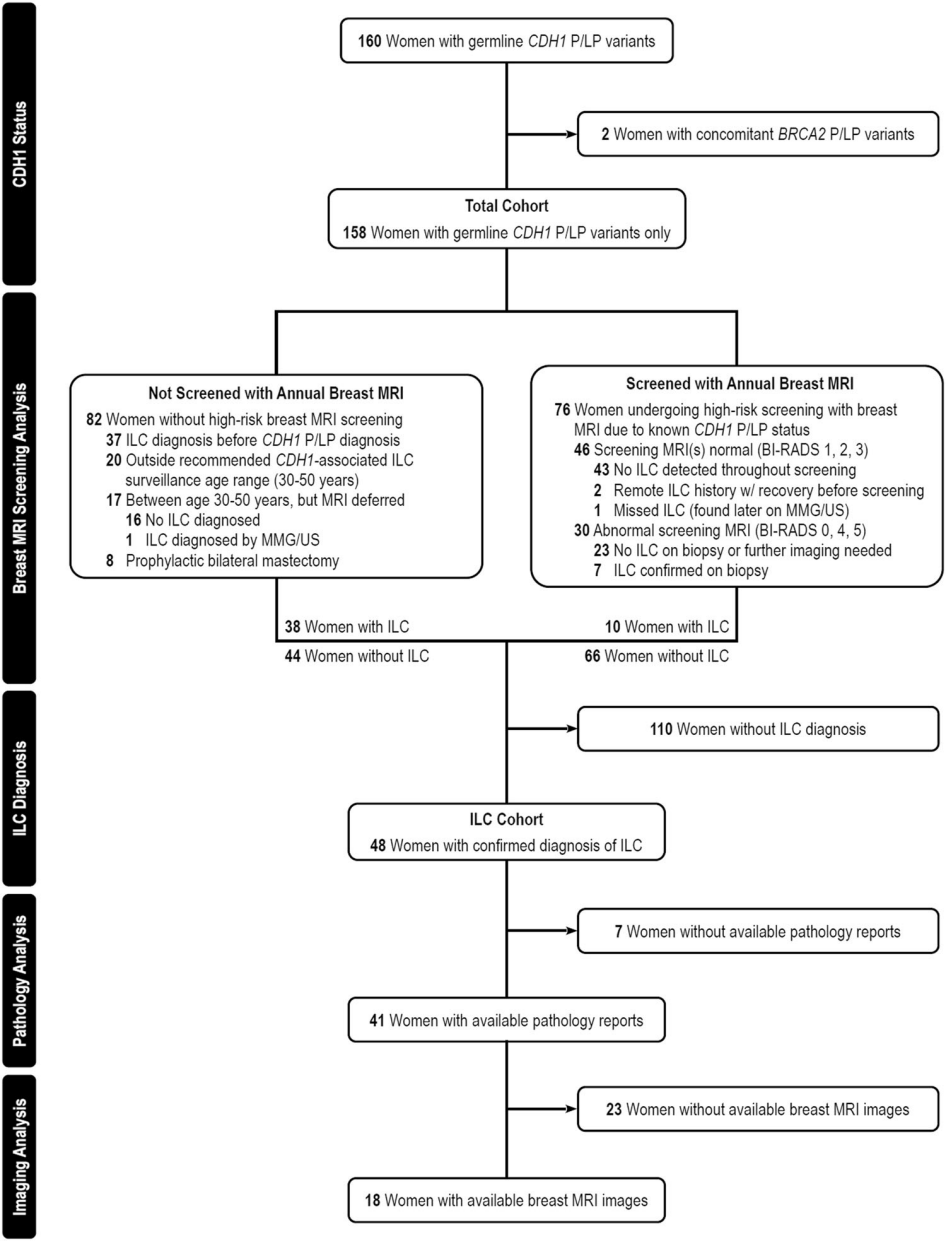# Author Correction: Defining features of hereditary lobular breast cancer due to *CDH1* with magnetic resonance imaging and tumor characteristics

**DOI:** 10.1038/s41523-023-00593-4

**Published:** 2023-10-23

**Authors:** Lauren A. Gamble, Paul H. McClelland, Martha E. Teke, Sarah G. Samaranayake, Paul Juneau, Amber L. Famiglietti, Andrew M. Blakely, Bernadette Redd, Jeremy L. Davis

**Affiliations:** 1 Surgical Oncology Program, Center for Cancer Research, National Cancer Institute, National Institutes of Health, Bethesda, MD USA; 2 Division of Library Services, Office of Research Services, National Institutes of Health, Bethesda, MD USA; 3 Radiology and Imaging Sciences, National Institutes of Health Clinical Center, Bethesda, MD USA

**Keywords:** Breast cancer, Cancer imaging

Correction to: *npj Breast Cancer* 10.1038/s41523-023-00585-4, published online 27 September 2023

In this article, the labels on the left-hand side of Figure 1 did not display properly. The original article has been corrected.