# Supplementary material for: Defining features of hereditary lobular breast cancer due to CDH1 with magnetic resonance imaging and tumor characteristics
Source: NPJ Breast Cancer. 2023 Sep 27;9:77. doi: 10.1038/s41523-023-00585-4 (PMC10533560; doi:10.1038/s41523-023-00585-4)

**Supplementary Table 1. Confusion matrix for breast MRI as a screening modality for ILC among women with *CDH1* pathologic variants.** Among 76 women screened with breast MRI, 30 (39%) had abnormal breast MRI findings (BI-RADS 0, 4, 5), and 22 of these abnormal MRIs had corresponding histopathology reports available for comparison.

| | | Actual (Biopsy) | | |
|---|---|---|---|---|
| | | **Positive** | **Negative** | |
| **Predicted (Breast MRI)** | **Positive** | 7 (TP) | 15 (FP) | 22 |
| | **Negative** | 1 (FN) | 45 (TN) | 46 |
| | | 8 | 60 | 68 |

Sensitivity = TP / (TP + FN) = 7 / 8 = 87.5%
Specificity = TN / (TN + FP) = 45 / 60 = 75.0%
PPV = TP / (TP + FP) = 7 / 22 = 31.8%
NPV = TN / (TN + FN) = 45 / 46 = 97.8%
FPR = FP / (FP + TN) = 15 / 60 = 25.0%
FNR = FN / (FN + TP) = 1 / 8 = 12.5%
FDR = FP / (FP + TP) = 15 / 22 = 68.2%
FOR = FN / (FN + TN) = 1 / 46 = 2.2%
Accuracy = (TP + TN) / (TP + FP + TN + FN) = 52 / 68 = 76.5%
MRI detection rate = TP / (total MRI scans) = 7 / 166 = 4.2%

MRI, magnetic resonance imaging
ILC, invasive lobular carcinoma
TP, true positive
TN, true negative
FP, false positive
FN, false negative
PPV, positive predictive value (precision)
NPV, negative predictive value
FPR, false-positive rate
FNR, false-negative rate
FDR, false-discovery rate
FOR, false-omission rate

**Supplementary Table 2.** Demographic and pathologic features of women with HLBC (study cohort) compared to ILC in the general population (SEER cohort)

| Variable | HLBC (*n* = 48) | ILC (SEER) (*n* = 100,266) | *p*-value |
|---|---|---|---|
| Age of breast cancer, years; median [IQR] | 47 [44-56] | n/a | n/a |
| 20-29 years | 0/48 (0.0) | 68/100,266 (0.1) | < 0.001 |
| 30-39 years | 5/48 (10.4) | 1,378/100,266 (1.4) | |
| 40-49 years | 21/48 (43.8) | 12,375/100,266 (12.3) | |
| 50-59 years | 14/48 (29.2) | 21,505/100,266 (21.4) | |
| 60-69 years | 7/48 (14.6) | 27,881/100,266 (27.8) | |
| 70-79 years | 1/48 (2.1) | 23,097/100,266 (23.0) | |
| 80+ years | 0/48 (0.0) | 13,962/100,266 (13.9) | |
| Histologic type | | | |
| Invasive lobular carcinoma, *n* (%) | 35/41 (85.4) | 99,612/100,266 (99.4) | < 0.001 |
| Mixed, n (%) | 6/41 (14.6) | 654/100,266 (0.6) | |
| Tumor size, cm; median [IQR] | 1.40 [0.80-1.80][a] | 2.00 [1.20-3.50][b] | 0.002 |
| ≤ 2 cm (T1), *n* (%) | 28/37 (75.7) | 40,617/79,287 (51.2) | 0.006 |
| > 2 and ≤ 5 cm (T2), *n* (%) | 4/37 (10.8) | 27,461/79,287 (34.6) | |
| > 5 cm (T3), *n* (%) | 5/37 (13.5) | 11,209/79,287 (14.1) | |
| Nuclear grade | | | |
| Low (grade 1), *n* (%) | 10/33 (30.3) | | n/a |
| Intermediate (grade 2), *n* (%) | 16/33 (48.5) | | |
| High (grade 3), *n* (%) | 7/33 (21.2) | | |
| Histologic grade | | | |
| Low (grade 1), *n* (%) | 7/30 (23.3) | 23,361/82,595 (28.3) | 0.776 |
| Intermediate (grade 2), *n* (%) | 19/30 (63.3) | 50,540/82,595 (61.2) | |
| High (grade 3), *n* (%) | 4/30 (13.3) | 8,694/82,595 (10.5) | |
| Ki-67 expression, *n* (%) | | | |
| < 10% positive (low), *n* (%) | 4/16 (25.0) | | n/a |
| ≥ 10 and < 20% positive (intermediate), *n* (%) | 8/16 (50.0) | | |
| ≥ 20% positive (high), *n* (%) | 4/16 (25.0) | | |
| ER positive, *n* (%) | 37/39 (94.9) | 91,473/94,517 (96.8) | 0.500 |
| PR positive, *n* (%) | 36/38 (94.7) | 75,820/93,475 (81.1) | 0.032 |
| HER2/neu positive, *n* (%) | 3/36 (8.3) | 2,908/56,156 (5.2) | 0.393 |
| Background LCIS presence, *n* (%) | 28/32 (87.5) | 1,068/100,266 (1.1) | < 0.001 |
| Background ALH presence, *n* (%) | 10/10 | n/a | n/a |
| Positive lymph nodes | 10/36 (27.8) | 30,721/85,210 (36.1) | 0.301 |
| 0 | 26 (72.2) | 54,489/85,210 (63.9) | 0.643 |
| 1-3 | 7 (19.4) | 17,986/85,210 (21.1) | |
| 4-9 | 1 (2.8) | 6,369/85,210 (7.5) | |
| 10+ | 2/36 (5.6) | 6,366/85,210 (7.5) | |

HLBC, hereditary lobular breast cancer
ILC, invasive lobular carcinoma
LCIS, lobular carcinoma in situ
ALH, atypical lobular hyperplasia
IQR, interquartile range
For continuous variables, data were described as medians with interquartile ranges [IQR, Q1-Q3]; these averages were compared via Mann-Whitney U test. Categorical variables were assessed via frequencies and proportions and were compared via χ2 test. All p-values were two-sided with a statistical significance evaluated at the 0.05 alpha level.
[a]Missing *n* = 11
[b]Missing *n* = 20,979

**Supplementary Figure 1. Trajectory graph of 75 women with *CDH1* pathologic variants undergoing surveillance breast MRI with correlating findings of abnormality and ILC diagnosis.** Open circle indicates *CDH1* diagnosis, closed circle indicates normal breast MRI, red diamond represents abnormal breast MRI and red "X" represents a diagnosis of ILC. White background represents the current age recommendations for breast cancer surveillance by International Gastric Cancer Linkage Consortium (IGCLC) of 30-50 years.

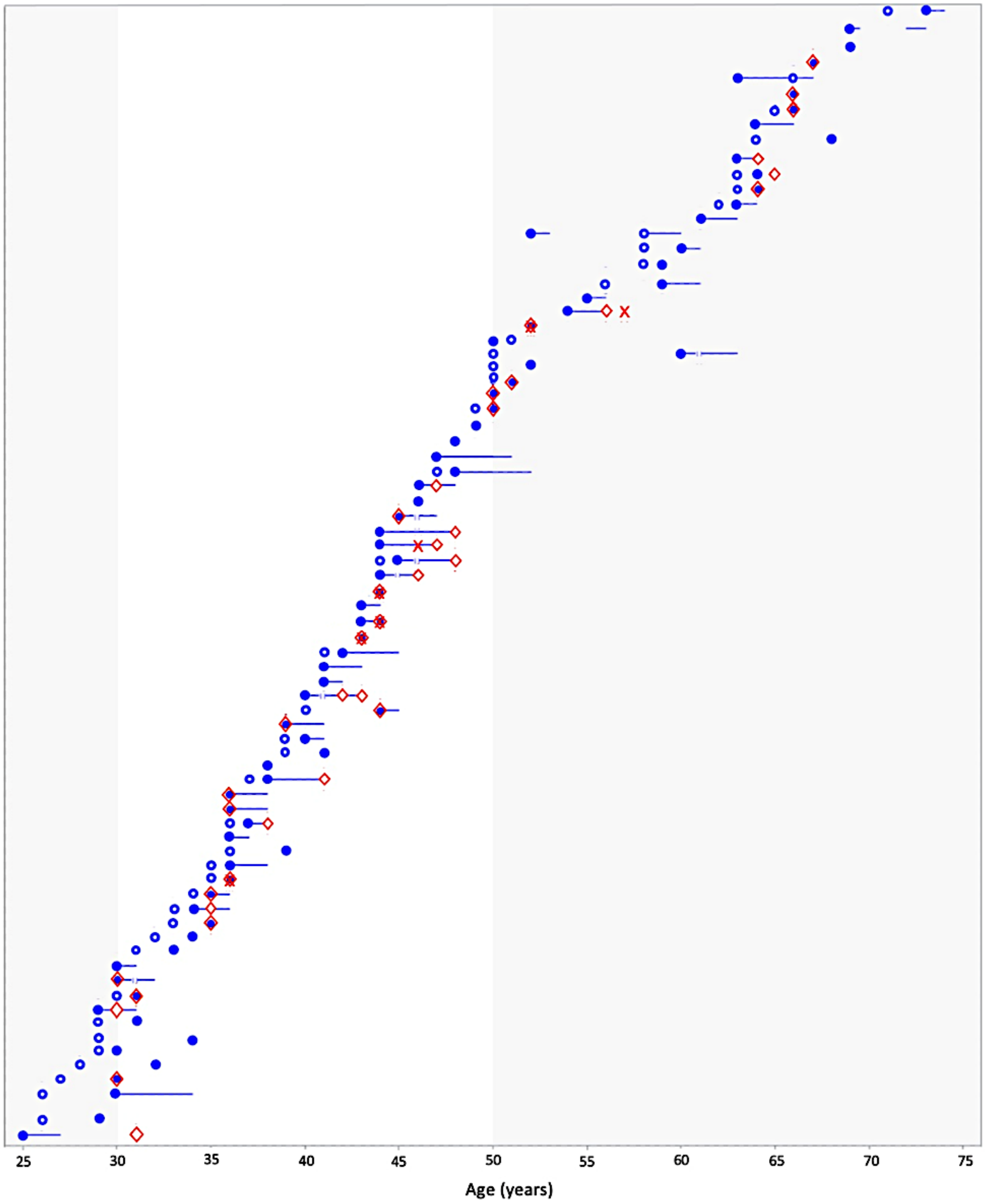

Supplement: Supplementary file 1 — Supplementary Files [file 41523_2023_585_MOESM1_ESM.pdf]
